# Supplementation With Bacillus clausii UBBC-07 Enhances Circulating Essential Amino Acids in Young Adults: A Double-Blind, Randomized, Controlled Trial

**DOI:** 10.7759/cureus.81119

**Published:** 2025-03-24

**Authors:** Mohamad Tarik, Lakshmy Ramakrishnan, Nidhi Bhatia, Atanu Roy, Devasenathipathy Kandasamy, Dinu S Chandran, Archna Singh, Mani Kalaivani, Jayanthi Neelamraju, Ratna S Madempudi

**Affiliations:** 1 Department of Cardiac Biochemistry, All India Institute of Medical Sciences, New Delhi, New Delhi, IND; 2 Department of Orthopaedics, All India Institute of Medical Sciences, New Delhi, New Delhi, IND; 3 Department of Physiology, All India Institute of Medical Sciences, New Delhi, New Delhi, IND; 4 Department of Radiodiagnosis, All India Institute of Medical Sciences, New Delhi, New Delhi, IND; 5 Department of Biochemistry, All India Institute of Medical Sciences, New Delhi, New Delhi, IND; 6 Department of Biostatistics, All India Institute of Medical Sciences, New Delhi, New Delhi, IND; 7 Centre for Research and Development, Unique Biotech Limited, Hyderabad, IND

**Keywords:** absorption, gut microbiome, nutrition, probiotic, probiotic supplementation, protein, sports medicine

## Abstract

Background and aim

Probiotics have been linked to improved gastrointestinal health and essential nutrient absorption. This study aimed to assess the impact of *Bacillus clausii*(*Shouchella clausii*) UBBC-07 plus whey protein supplementation on the bioavailability of circulating essential amino acids (EAAs) in physically active young adults.

Methods

In this double-blind, randomized, controlled trial, 70 physically active male participants (21.46±3.19 years) were instructed to ingest either a probiotic supplement containing two billion colony-forming unit (CFU) *Bacillus clausii* UBBC-07 + 20 g of whey protein or a control supplement containing placebo + 20 g of whey protein once daily for 60 days. All the participants followed a supervised exercise protocol. The circulating amino acid levels were determined using a high-performance liquid chromatography with fluorescence detection (HPLC-FLD) assay and compared using the student's t-test and a repeated measures analysis of variance (ANOVA).

Results

After 60 days, a significant improvement in the probiotic group was observed compared to the control group in terms of total levels of circulating EAAs (mean change: 258 pmol/μl, 95% CI: 161.5-354.4 vs. 76.4 pmol/μl, 95% CI: 16.5-136.4; p=0.002) and branched-chain amino acids or BCAAs (mean change: 144.2 pmol/μl, 95% CI: 89-199.3 vs. 37.5 pmol/μl, 95% CI: 7.3-67.8; p=0.001) as well as levels of isoleucine (p=0.003), leucine (p>0.001), and valine (p=0.001). Total plasma free amino acids (PFAAs) were also increased in the probiotic group (p<0.001). The improvement in the one-repetition maximum (RM) leg press was higher in the probiotic group as compared to the control group (mean change: 20.46 kg, 95% CI: 14.73-26.19 vs. 14.09 kg, 95% CI: 8.44, 19.73; p=0.045). A trend towards improvement in deadlift and vertical jump was also observed in the former group. No probiotic-mediated gastrointestinal upsets and respiratory symptoms or any other adverse events were observed.

Conclusion

A significant improvement in circulating EAA levels in the probiotic group suggests an enhancement of protein absorption with *Bacillus clausii* UBBC-07 supplementation. The effect of BCAAs, which enhance muscle strength, is evident in the significant improvement in leg press and a trend towards improvement in deadlift and vertical jump in the probiotic group. This has positive implications for individuals involved in sports activities.

## Introduction

The World Health Organization (WHO) and the United Nations Food and Agriculture Organization (FAO) define probiotics as live microorganisms that provide health benefits to the host when administered in adequate amounts [1]. They confer several health benefits by modulating host microbiota. Improving digestion and strengthening the immune system are the primary ways in which probiotics exert their effects [2,3]. In sportspersons, the diversity of the gut microbiome is impacted due to intestinal hypoperfusion [4]. Probiotic supplementation may help overcome the negative effects of strenuous exercise in such individuals as they increase the bioavailability of nutrients that are disturbed due to endurance exercise. A limited number of studies have demonstrated improved amino acid absorption with probiotic supplementation [5-7]. The effect of probiotics is strain-specific [8], and in this study, we explored the beneficial effects of supplementation with *Bacillus clausii* UBBC-07 and whey protein.

Amino acids are the building blocks of proteins, which are essential for tissue growth, repair, and maintenance. They are involved in metabolic pathways, and provide energy, especially during prolonged exercise or fasting [9]. Individuals who are involved in sports and other physical activities may require additional dietary protein, particularly essential amino acids (EAAs) and more specifically, branched-chain amino acids (BCAAs) including leucine, isoleucine, and valine. They promote muscle growth, aid in recovery and tissue repair, and enhance both physical and mental responses [10]. Unlike non-essential amino acids, the body's inability to produce EAAs highlights the importance of maximizing their intake from dietary protein. Whey protein is a mixture of protein isolated from the liquid material created as a by-product of cheese production. It contains all the nine EAAs and is especially rich in BCAAs which play a major role in muscle protein synthesis [9]. Previous studies reported that whey protein enhances the body’s protein recovery and synthesis after exercise, reducing soreness and fatigue [11]. It gets digested quickly compared to other whole food protein sources (e.g., chicken, eggs, beans), which makes it ideal for post-workout nutrition. It is also considered safe to consume as per the European Food Safety Authority [12]. Protein absorption can be improved through the addition of a probiotic along with whey protein.

The use of *Bacillus clausii* as a probiotic supplement is common, especially when administered alongside antibiotics, as it helps enhance the intestinal flora [13]. *Bacillus clausii* has been reclassified as *Shouchella clausii*. Before that, it was known as *Alkalihalobacillus clausii*. However, it is still commonly referred to by the older name; hence, the specific strain in this study shall be referred to as *Bacillus clausii* UBBC-07.

*Bacillus clausii *UBBC-07, being a spore-forming probiotic strain, can survive and germinate in challenging environments and is considered a safe probiotic for humans who are not immune compromised [14-16]. Studies conducted with *Bacillus clausii *UBBC-07 have indicated its effectiveness in treating acute diarrhea in both adults and children [17,18] and in alleviating symptoms of upper respiratory tract infections (URTIs) in children [19]. A recent study reported that it releases an extracellular protein/protease, which has prominent antimicrobial activity against *Bacillus cereus* and *Salmonella enterica *(diarrhea-causing pathogens) [20]. The effect of the UBBC-07 strain on protein absorption has not yet been investigated. We hypothesized that *Bacillus clausii* supplementation may enhance protein absorption by promoting gut health, increasing digestive enzyme activity, and modulating gut microbiota, thereby leading to increased bioavailability of EAAs and their utilization. Hence, we assessed the impact of *Bacillus clausii *UBBC-07 consumed in combination with whey protein on circulating EAAs and muscle strength in those involved in sports.

## Materials and methods

Study design

This randomized, double-blind, controlled study was conducted to evaluate the impact of a 60-day supplementation regimen of *Bacillus clausii* UBBC-07 along with 20 g of whey protein on plasma free amino acid (PFAA) levels in young participants (18-28 years) involved in sports activities. This study was conducted from May 2018 to December 2019 at the All India Institute of Medical Sciences (AIIMS), New Delhi, India. It was approved by the Institutional Ethical Committee (IEC) at AIIMS (ref no. IEC-595/05.01.2017) and was registered in the Clinical Trials Registry of India (CTRI No: CTRI/2017/03/008117). All participants signed an IEC-approved informed consent form before beginning the study.

Study participants

Healthy subjects (n=70) between 18 to 28 years of age who were involved in performing regular exercise in the gym, running, or actively participating in games like cricket and football in the Delhi/National Capital Region (NCR), India, were included in the study. A health history questionnaire was administered to determine study eligibility. Subjects who were excluded included those with a history of any gastrointestinal disease, lactose intolerance, and other serious medical conditions, those who had taken laxatives, antibiotics, probiotics/prebiotics (including yogurt and lacto-fermented beverages) within the 60 days before screening/enrolment, and those who had donated more than 200 ml of blood three months before the start of the study.

Intervention

The eligible participants randomized to the probiotic group ingested two billion colony-forming units (CFU) of *Bacillus clausii *UBBC-07 (Patent ID: MTCC5472, Unique Biotech Limited, India) [16] and 20 g whey protein (CN: 35022091, Polmlek Group, Warszawa, Poland; 80% whey protein concentrate (WPC80), amounting to 15.4 g protein and 4.6 g other component such as lactose, other carbohydrates and fat etc.) once daily for 60 days. Participants randomized to the control group ingested 20 g of whey protein along with a placebo. The intervention and control supplements were chocolate flavored. To maintain its double-blind nature, all the study materials were packed into individual plastic sachets of same color and size with non-identifying numbers and letters by a person not involved in the trial. The sachets were provided by the funding agency Unique Biotech Limited. Each sachet was dissolved in 500 ml of normal water before ingestion. The participants were advised to drink the fortified water within 30 min and at least one hour before dinner.

Exercise protocol for the duration of study

All the participants followed the same exercise protocol four times per week for 60 days under the supervision of a physiotherapist (Appendix A). The exercise protocol consisted of strength (one to five repetition maximum or RM loads with three to five minutes rest) and hypertrophy (eight to 12 RM loads with 60 seconds rest). The first training day was allotted for overall muscle strength, the second training day for back and biceps muscle strength, the third training day for leg muscle strength, and the fourth training day was assigned for chest-shoulders-triceps muscle strength. One day each was allotted for strength and hypertrophy of the various muscle groups. Subjects in both arms of the study maintained the exercise regimen during the study period.

Sample size

No appropriate information was available from previous studies for sample size calculation. Based on the fact that there would be a clinically meaningful improvement in plasma amino acid levels of the experimental group (expected to be approximately 15%) in comparison with the placebo group (244.6±52.8 µmol/L, obtained from a previous study [21], the estimated sample size was 32 subjects after applying an α-error of 5% and a power of 80%. After considering ~10% expected dropouts, 35 subjects were recruited in each group.

Randomization, allocation, and blinding

Study participants were randomized by a simple randomization process using MS Excel (Microsoft Corp., Redmond, WA, US) and a random table generated on the computer by an independent statistician. Each subject was assigned a unique identifier, and the computer-generated random numbers were used to allocate them into study groups, ensuring equal and fair distribution. Participants were enrolled by the research staff and randomly allocated (1:1 ratio) to either probiotic group or control group after assessment according to the inclusion and exclusion criteria. In order to maintain the double-blind nature of the study, the assignments were prepared in serially numbered, sealed, opaque, envelopes by a person not involved in the trial. Sachets of the same color and size were used to pack the powdered supplement for all the groups. The entire research staff and participants were blinded to the randomization results until after the initial analysis.

Dietary monitoring

Before starting the supplementation, a dietary log was customized by a dietician and provided to each study subject to facilitate diet replication during the study period. A subject-specific calorie requirement was calculated using the Mifflin-St Jeor formula [22]. An intake of 1.4 g protein/kg body weight was maintained with diet and 20 g of whey protein (containing 15.4 g protein). The macronutrients in the diet were controlled to 50% carbohydrates, 25% protein, and 25% fat.

Primary and secondary outcome measures

The primary outcome measure was the total level of EAAs. The secondary outcome measures included the total and individual levels of BCAAs (leucine, isoleucine and valine), and the total and individual levels of other essential and non-essential amino acids including alanine, arginine, asparagine, aspartic acid, cysteine, glutamic acid, glutamine, glycine, histidine, hydroxyproline, lysine, methionine, phenylalanine, proline, serine, threonine, tryptophan, and tyrosine. We also assessed markers of muscle strength (leg press, bench press, deadlift, and vertical jump), and gastrointestinal and upper respiratory symptoms as secondary outcomes. All primary and secondary outcome measures were assessed at baseline (0 day), at the mid-point (30 days) and at study completion (60 days).

Demographics and anthropometric measurements

All randomized participants reported to the laboratory in the morning after eight to 10 hours of fasting before starting the supplement. The height, weight, body mass index (BMI), waist circumference (WC), hip circumference (HC), heart rate, systolic blood pressure (SBP), diastolic blood pressure (DBP), and heart rate of the participants were recorded.

Biochemical analysis

After recruitment, 5 ml of venous blood sample was collected from the eligible participants at baseline in a ethylenediamine tetraacetic acid (EDTA)-anticoagulated tube (for plasma) and in a plain tube (for serum). The mid-point (30 days) and 60-day blood samples were collected within ±2 days. Plasma and serum were separated and stored at -80°C prior to estimation of PFAAs and other biochemical parameters.

Analysis of PFAAs

Analysis of amino acids was performed by a manual pre-column derivatization process on reverse-phase HPLC assay (1260 Infinity HPLC system, Agilent Technologies, CA, USA) using fluorescence detection. Briefly, 300 μl of 3% sulfosalicylic acid was mixed with 200 μl plasma samples to precipitate protein and centrifuged at 15000g for 10 min at 4°C. The supernatant was filtered through a syringe filter (0.22 µm). In the derivatization process, a 2.0 µl sample was mixed with 5 µl borate buffer (P/N: 5061-3339; Agilent Technologies, CA, USA), and after 30 sec, 2 µl ortho-phthalaldehyde or OPA (P/N: 5061-3335; Agilent Technologies, CA, USA) and 1 µl 9-fluorenyl-methyl chloroformate or FMOC (P/N: 5061-3337; Agilent technologies, CA, USA), and 30µl injection diluents (100 mL of mobile phase A + 0.4 mL con. H_3_PO_4_) were added and mixed 10 times smoothly. Twenty microliters of this reaction mixture was injected and the separation of amino acids was performed by reverse phase C18 column (Poroshell 120 HPH-C18; 4.6 mm X 100 mm X 2.7 µm; P/N: 695972-702, Agilent Technologies, CA, USA) in gradient program of mobile phase A (1.4 g Na_2_HPO_4_.10H_2_O + 3.8 g Na_2_B_4_O_7_ in 1 L Milli-Q Water, pH 8.2) and mobile phase B (Acetonitrile: Methanol: H_2_O (45: 45: 10) starting with 2% of B from 0 min, gradient of 2% to 100% of B from 0.35 min to 15.4 min, 100% of B from 15.4 to 18.7 min, and 2% of B from 18.8 min to 20 min at the constant flow rate of 1.2 ml/min. Two different transition points were used to detect OPA (excitation: 340 nm, emission: 450 nm) and FMOC (excitation: 260 nm, emission: 325 nm) derivatized amino acids in a single chromatogram using the timetable function (wavelength switched between lysine and hydroxyproline peak) of the fluorescence detector. Individual amino acids were quantified against a standard curve of the respective amino acid with five different concentrations (range: 900-9 pmol/μl). The precision of the assay was calculated as the inter-day and intra-day coefficient of variation (CV), and the accuracy of the assay was determined as the recovery percentage.

Assessment of muscle strength

Muscle strength was determined at baseline and at study end by one RM of leg press, bench press, and deadlift. For the vertical jump, the subjects were instructed to jump upwards into the air to a maximum height three times. The final vertical jump was calculated by subtracting the subject’s height with the arm extended upward in the standing position from the maximum height of all the three jumps.

Assessment of safety and adverse events

To assess the safety and adverse events of probiotic supplementation, symptoms of gastrointestinal upset and upper respiratory infection were evaluated at baseline and at the end of the study using the Gastrointestinal Symptom Rating Scale (GSRS) [23] and Wisconsin Upper Respiratory Symptom Survey (WURSS) [24] questionnaires on a daily basis. Additional adverse events were also obtained through a spontaneous report to a health assistant by the participants.

Statistical analysis

Data was entered and managed in MS Excel and statistical analysis was carried out using IBM SPSS Statistics for Windows, Version 24 (Released 2016; IBM Corp., Armonk, New York, United States). Categorical data was summarized as frequency and percentage (%) and compared among the two groups using Fisher’s exact test. Data of continuous variables were assessed for normality and summarized as mean ± standard deviation (SD) or median (min-max) as appropriate. Variables that followed a normal distribution were analyzed using the Student’s t-test for independent samples, and the two-way repeated measures ANOVA to assess the effect of time and group, between probiotic and placebo group for intention-to-treat (ITT) analysis (n=35) and per-protocol (PP) analysis (n=28) in each group. Variables that did not follow a normal distribution were analyzed by the Wilcoxon rank-sum test and the Freidman test. Missing values for the ITT analysis were handled using the selective multiple imputation method. The randomization codes were broken after completing the statistical analysis of a randomly-allocated dataset. P values <0.05 were reported as statistically significant. 

## Results

**Figure 1:**
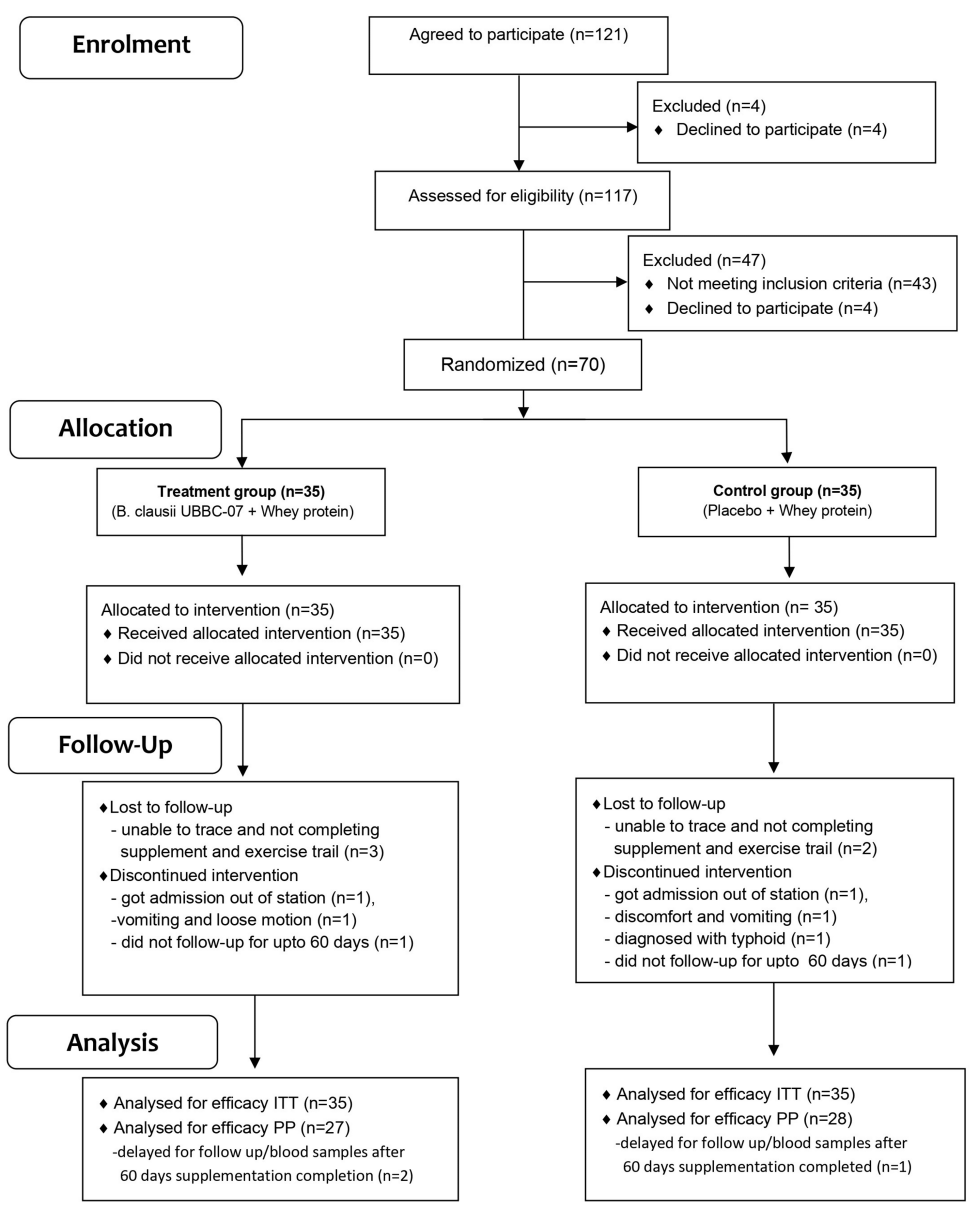
A Consolidated Standards of Reporting Trials (CONSORT) flow diagram CONSORT 2010 statement [25] ITT: Intention-to-treat; PP: per-protocol.

In this double-blind randomized controlled trial, participants (n=121) were screened between 2018 and 2019. Seventy subjects (mean±SD: 21.46±3.19 years) were found to be eligible for the study as per the inclusion and exclusion criteria. They were randomized into two groups (n=35 in each group). During the follow-up, several subjects (~20%) dropped out of the study for various reasons (Figure 1).

Out of the 35 subjects, the prescribed study protocol was completed in all respects by 27 subjects in the probiotic group and by 28 subjects in the control group. Their demographic and anthropometric details are depicted in Table 1.

**Table 1:** Demographic characteristics of the participants Data are represented as mean±SD and compared between the probiotic and control groups by the Student’s t-test. A p value <0.05 is considered significant. BMI: body mass index; WC: Waist circumference; HC: Hip circumference; SBP: Systolic blood pressure; DBP: Diastolic blood pressure.

Variables	*Bacillus clausii* UBBC-07 (n=35)	Control (n=35)	t-value	p-value
Age (years)	22±3.25	20.91±3.08	1.43	0.156
Weight (kg)	64.51±10.24	64.3±10.27	0.084	0.933
Height (cm)	167.99±4.89	169.01±5.55	-0.81	0.420
BMI (kg/m^2^)	22.91±3.81	22.46±3.1	0.54	0.591
WC (cm)	81.79±9.59	81.81±8.18	-0.01	0.991
HC (cm)	94.41±8.1	93.96±6.48	0.25	0.801
Heart rate (per min)	82.69±10.8	83.2±13.4	-0.18	0.860
SBP (mmHg)	122.43±10.89	123.93±11.42	-0.56	0.576
DBP (mmHg)	77.23±8.6	77.26±8.84	-0.14	0.989

No significant differences in age, weight, height, BMI, WC, HC, heart rate, SBP, and DBP were observed between the groups at baseline and after the intervention of 60 days.

Effect of probiotic supplementation on total PFAAs

The analytical performance of the HPLC-FLD assay for the analysis of amino acids was evaluated based on the key validation parameters. This method exhibited excellent linearity, as indicated by the high coefficient of determination (R²>0.995 for all 21 amino acids) over the five-point calibration curve. The intra-day CV was less than 7.2% and the inter-day CV was less than 9.8% for all amino acids, which indicated that the assay was highly precise with acceptable recovery (99%, range: 96 to 104%) for all the amino acids.

A two-way repeated measures ANOVA was performed to evaluate the impact of probiotic supplementation on PFAAs over three time points: baseline, 30 days and 60 days (Table 2 with the ITT analysis and Appendix B with the PP analysis).

**Table 2:** Levels of plasma free amino acids (PFAAs) between groups of physically active adult males after supplementation (ITT analysis) Data are represented as mean±SD and compared by repeated measures ANOVA between the probiotic and control groups. A p value <0.05 is considered significant. *B. clausii: Bacillus clausii; *ITT: Intention-to-treat; BCAAs: Branched-chain amino acids; EAAs: Essential amino acids.

Amino acids (pmol/μl)	Group	Baseline	30 days	60 days	Time		Group		Time x Group	
					F-value	p-value	F-value	p-value	F-value	p-value
Total PFAAs	B. clausii	3936±1074	5111±1463	5128±1192	16.9	<0.001	9.1	0.004	8.9	<0.001
	Control	3949±1097	4125±928	4148±839						
Total BCAAs	B. clausii	397±99	581±266	541±172	20.1	<0.001	4.6	0.035	5.9	0.007
	Control	412±107	470±125	449±90						
Total EAAs	B. clausii	857±215	1183±443	1115±316	23.3	<0.001	3.6	0.063	5.7	0.007
	Control	884±201	1000±253	960±168						
Aspartic acid	B. clausii	9.2±2.7	15±6.5	13.8±5.4	10.4	<0.001	2.6	0.109	13.5	<0.001
	Control	11.1±5.2	9.9±2.9	12.4±6.5						
Glutamic acid	B. clausii	66.7±46.3	106.7±57.2	94.5±49.1	14.1	<0.001	3.9	0.051	2.3	0.109
	Control	63.5±28.8	80.5±44.8	74.7±28.1						
Asparagine	B. clausii	65.1±21.2	81.8±28.7	78.5±28.8	19.9	<0.001	1.7	0.194	1.0	0.382
	Control	62.8±17	73.6±15.2	71.2±14.9						
Serine	B. clausii	124±39	176±66	163±52	13.2	<0.001	2.1	0.155	8.4	0.001
	Control	137±45	142±42	144±38						
Glutamine	B. clausii	1391±475	1694±531	1840±522	7.2	0.001	12.5	0.001	10.4	<0.001
	Control	1341±525	1241±413	1316±445						
Histidine	B. clausii	84.2±23.7	110.9±33.1	107.2±26.8	17.0	<0.001	1.2	0.273	7.6	0.001
	Control	91.3±21.4	95.6±28.5	97.6±24.8						
Glycine	B. clausii	235±127	289±166	280±151	2.5	0.089	1.0	0.325	0.6	0.58
	Control	231±129	253±124	243±129						
Threonine	B. clausii	161±52	244±91	239±86	25.5	<0.001	2.5	0.12	7.4	0.001
	Control	177±54.5	211±76	191±59						
Arginine	B. clausii	76.2±25.7	88.6±48.6	109.1±44.6	17.7	<0.001	5.2	0.025	3.6	0.031
	Control	73.3±24.5	67.4±26.4	86.2±32.6						
Alanine	B. clausii	481±176	646±315	621±193	10.7	<0.001	3.5	0.066	1.9	0.332
	Control	458±141	542±255	534±154						
Tyrosine	B. clausii	62.4±14.8	77.5±20.9	81±22	22.1	<0.001	7.2	0.009	3.0	0.058
	Control	60.1±11	67±15.9	68.7±13.2						
Cysteine	B. clausii	277±131	378±181	419±217	5.9	0.004	6.1	0.016	4.6	0.011
	Control	290±152	285±97	302±130						
Valine	B. clausii	260±63	374±164	361±134	19.0	<0.001	5.0	0.029	6.7	0.003
	Control	268±77	301±84	290±65						
Methionine	B. clausii	20.7±4.2	26±8.7	26±6.2	12.8	<0.001	2.6	0.115	9.3	<0.001
	Control	22.2±3.3	22.3±5	23±3.9						
Tryptophan	B. clausii	39.1±7.7	46.1±12.5	47.7±14.1	9.2	<0.001	1.7	0.200	4.3	0.017
	Control	40.9±7.7	42±8.3	42.7±7.4						
Phenylalanine	B. clausii	41.7±8.4	51.4±12.9	51.7±10.9	17.0	<0.001	2.9	0.093	3.1	0.050
	Control	42.4±7.9	46.4±11.7	46.5±8.4						
Isoleucine	B. clausii	40.5±11.7	63.5±32.7	61.2±22.7	23.2	<0.001	5.8	0.018	6.4	0.002
	Control	42.1±9.5	48.8±10.8	49.1±12.3						
Leucine	B. clausii	97±29	161±90	143±42	16.7	<0.001	10.2	0.002	6.5	0.006
	Control	101±27	118±33	108±26						
Lysine	B. clausii	112±90	139±102	130±81	3.6	0.03	1.4	0.274	0.1	0.909
	Control	98±54	119±73	111±52						
Hydroxyproline	B. clausii	60.4±43.2	72.7±26.6	81.8±38.3	2.7	0.088	0.12	0.731	2.1	0.14
	Control	68.7±38.7	70.6±23.3	69.9±23.2						
Proline	B. clausii	232±110	337±219	352±167	9.5	<0.001	1	0.348	5.8	0.006
	Control	270±159	272±108	292±117						

The ITT analysis revealed a significant main impact for time (F (2, 136)=16.9; p<0.001) and group (F (1, 68)=9.1; p=0.004) for total PFAAs, and a significant interaction between time and group (F (2, 136)=8.9; p<0.001).

A significant interaction between time and group was observed for total EAAs (p=0.007) and total BCAAs (p=0.007) as well as individual BCAAs including isoleucine (p=0.002), leucine (p=0.002) and valine (p=0.003) and other individual essential amino acids including histidine (p=0.001), threonine (p=0.001), methionine (p<0.001), tryptophan (p=0.017), and phenylalanine (p=0.050) in the ITT analysis. However, no time and group interactions were seen for lysine (p=0.909). A significant main impact of time was revealed for all these total and individual EAAs (p<0.001). A significant impact of group was observed for total BCAAs (p=0.035) and the three individual BCAAs, isoleucine (p=0.018), leucine (p=0.002) and valine (p=0.029). However, there was no significant main effect for group for total EAAs (p=0.063), histidine (p=0.273), threonine (p=0.120), methionine (p=0.115), tryptophan (p=0.200), and phenylalanine (p=0.093), but the tendency for increment was higher in the probiotic group.

Among the non-essential amino acids, time by group interaction was observed for aspartic acid (p<0.001), serine (p=0.001), glutamine (p<0.001), arginine (p=0.031), cysteine (p=0.011), proline (p=0.006), whereas none was observed for glutamic acid (p=0.109), asparagine (p=0.382), glycine (p=0.580), alanine (p=0.340), tyrosine (p=0.058), and hydroxyproline (p=0.140). A significant main impact for time was revealed for all these non-essential amino acids except glycine (p=0.089) and hydroxyproline (p=0.088). The main effect for group was seen for glutamine (p=0.001), arginine (p=0.025), tyrosine (p=0.009), and cysteine (p=0.016).

Change in amino acid levels

**Figure 2:**
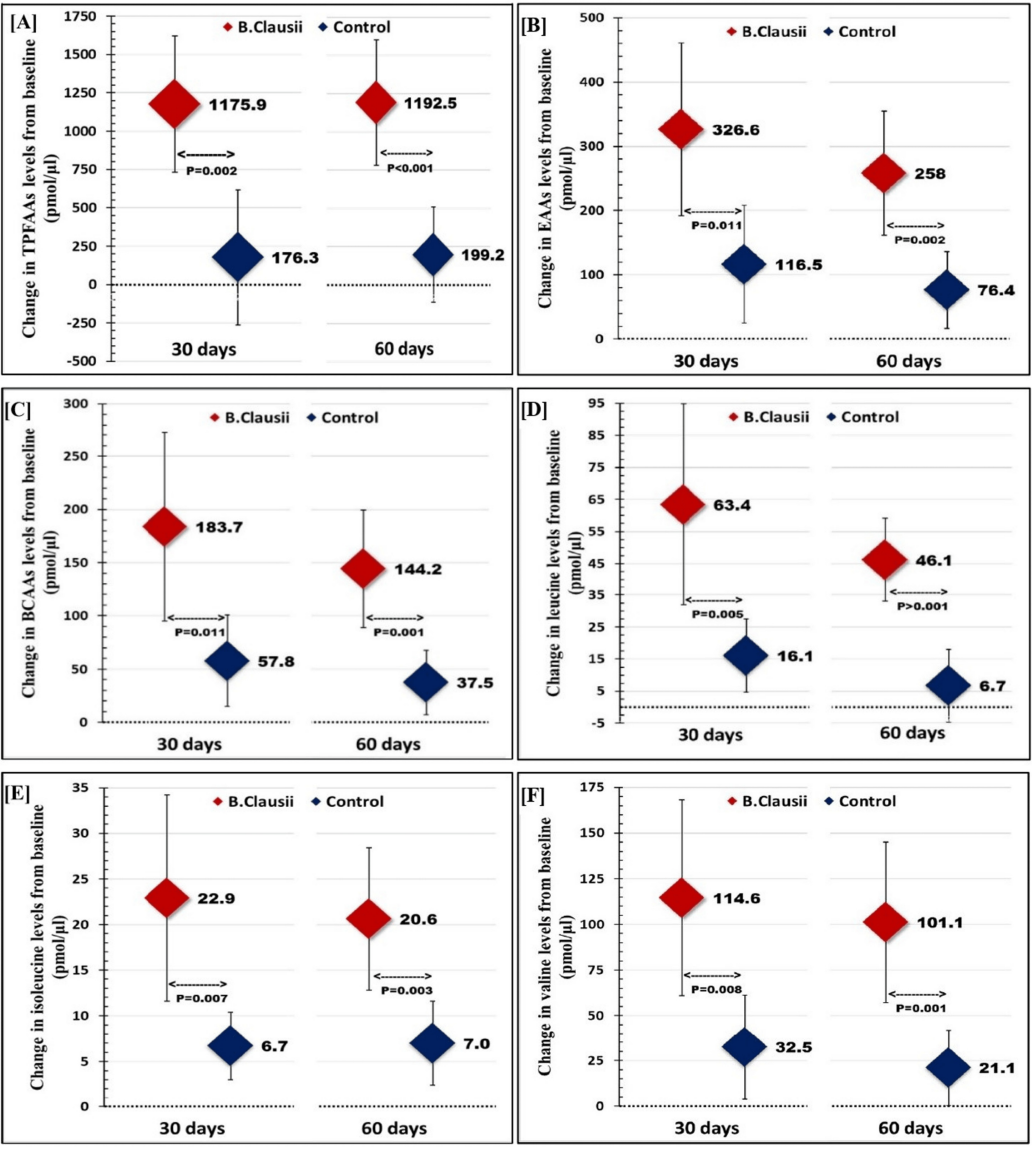
Change from baseline in circulating amino acid levels between the probiotic and control groups Mean change in circulating amino acid levels from baseline at 30 days and 60 days of supplementation and compared using Student’s t-test between the probiotic and control groups. A p value <0.05 was considered significant. The findings showed that the changes in total PFAAs (Figure 2A), total EAAs (Figure 2B), total BCAAs (Figure 2C) and individual BCAAs including leucine (Figure 2D), isoleucine (Figure 2E), and valine levels (Figure 2F) were significantly greater in the probiotic group as compared to the control group. *B. clausii: Bacillus clausii; *TFAAs: total plasma free amino acids; EAAs: essential amino acids; BCAAs: branched-chain amino acids.

The change from baseline in total PFAAs, EAAs, and BCAAs levels is depicted in Figure 2. 

After 60 days of intervention, when compared with the baseline, the change in total PFAAs (mean difference: 1192.5 pmol/μl, 95% CI: 782.1-1602.8 vs. 199.2 pmol/μl, 95% CI: -111.3-509.6; p<0.001), EAAs (mean difference: 258 pmol/μl, 95% CI: 161.5-354.4 vs. 76.4 pmol/μl, 95% CI: 16.5-136.4; p=0.002), and BCAAs (mean difference: 144.2 pmol/μl, 95% CI: 89-199.3 vs. 37.5 pmol/μl, 95% CI: 7.3-67.8; p=0.001) was significantly more pronounced in the probiotic group as compared to the control group.

After 30 days, the total PFAAs, EAAs and BCAAs were increased by 25.4% (29.9% vs. 4.5%), 24.9% (38.1% vs. 13.2%) and 32.3% (46.3% vs. 14.0%), and similarly after 60 days, the total PFAAs, EAAs and BCAAs were increased by 25.3% (30.3% vs 5.0%), 21.5% (30.1% vs 8.7%) and 27.2% (36.3% vs 9.10%) in the probiotic group as compared to the control group, respectively. At the end of 60 days, individual BCAAs such as isoleucine improved by 34.5% (51.1% vs. 16.6%; p=0.017), leucine by 40.9% (47.5% vs. 6.6%, p=0.001) and valine by 31% (38.9% vs. 7.9%, p=0.029) in the probiotic group as compared to the control group. The improvement in amino acids levels was higher at 30 days in comparison to 60 days of intervention.

Effect of *Bacillus clausii* UBBC-07 supplementation on muscle strength

The effect on muscle strength was assessed by measuring one RM dead lift, bench press, leg press and vertical jump (Appendix C). A significant impact for time was observed for all (p<0.001) but no significant impact of group was revealed for these markers. We observed a significant interaction between time and group only for vertical jump (p=0.044) but there was no interaction for the dead lift, bench press, and leg press. When we compared the change from baseline, the improvement in one RM leg press was higher in the probiotic group as compared to control group (mean difference: 20.46 kg, 95% CI: 14.73-26.19 vs. 14.09 kg, 95% CI: 8.44-19.73; p=0.045). A trend of improvement was observed in the probiotic group as compared to the control group for one RM deadlift (mean difference: 14.79 kg, 95% CI: 9.91-19.67 vs. 9.92 kg, 95% CI: 6.61-13.23; p=0.141), and vertical jump (mean difference: 7.78 cm, 95% CI: 5.49-10.07 vs. 4.65 cm, 95% CI: 2.50-6.79; p=0.109) respectively.

Safety measures and adverse events

The effect of *Bacillus clausii* supplementation on gastrointestinal upsets and upper respiratory symptoms are summarized in Table 3.

**Table 3:** Effect on gastrointestinal upsets and upper respiratory symptoms between the Bacillus clausii UBBC-07 and control groups Data are represented as number (percentage). 'No' represents do not have symptoms or scored 0 in scale in the GSRS/WURSS-21 scale questionnaires. A p value <0.05 is considered significant. 'Yes' represents have symptoms or scored ≥1 in the GSRS/WURSS-21 scale questionnaires. Compared with Fisher's exact test. GSRS: Gastrointestinal symptom rating scale; WURSS: Wisconsin upper respiratory symptom survey.

Variable			Bacillus clausii	Control	Chi-square value	p-value
Gastrointestinal upsets						
Abdominal pains		No	25 (92.6)	25 (89.3)	0.180	0.999
		Yes	2 (7.4)	3 (10.7)		
Heart burn		No	25 (92.6)	28 (100)	2.150	0.236
		Yes	2 (7.4)	0 (0)		
Acid regurgitation		No	25 (92.6)	27 (96.4)	0.392	0.611
		Yes	2 (7.4)	1 (3.6)		
Sucking sensations in the epigastrium		No	25 (92.6)	26 (92.9)	0.001	0.999
		Yes	2 (7.4)	2 (7.1)		
Nausea and vomiting		No	26 (96.3)	27 (96.4)	0.001	0.999
		Yes	1 (3.7)	1 (3.6)		
Borborygmus		No	25 (92.6)	27 (96.4)	0.392	0.611
		Yes	2 (7.4)	1 (3.6)		
Abdominal distension		No	25 (92.6)	27 (96.4)	0.392	0.611
		Yes	2 (7.4)	1 (3.6)		
Eructation		No	26 (96.3)	26 (92.9)	0.315	0.999
		Yes	1 (3.7)	2 (7.1)		
Increased flatus		No	27 (100)	28 (100)	-	-
		Yes	0 (0)	0 (0)		
Decreased passage of stools		No	25 (92.6)	27 (96.4)	0.392	0.611
		Yes	2 (7.4)	1 (3.6)		
Increased passage of stools		No	23 (85.2)	27 (96.4)	2.10	0.193
		Yes	4 (14.8)	1 (3.6)		
Loose stools		No	25 (92.6)	25 (89.3)	0.182	0.999
		Yes	2 (7.4)	3 (10.7)		
Hard stools		No	25 (92.6)	24 (85.7)	0.669	0.669
		Yes	2 (7.4)	4 (14.3)		
Urgent need for defecation		No	27 (100)	28 (100)	-	-
		Yes	0 (0)	0 (0)		
Feeling of incomplete evaculation		No	26 (96.3)	27 (96.4)	0.001	0.999
		Yes	1 (3.7)	1 (3.6)		
Respiratory symptoms						
How sick do you feel today	No		23 (85.2)	26 (92.9)	0.832	0.422
	Yes		4 (14.8)	2 (7.1)		
Runny nose	No		24 (88.9)	24 (85.7)	0.125	0.999
	Yes		3 (11.1)	4 (14.3)		
Plugged nose	No		25 (92.6)	26 (92.9)	0.001	0.999
	Yes		2 (7.4)	2 (7.1)		
Sneezing	No		23 (85.2)	26 (92.9)	0.832	0.422
	Yes		4 (14.8)	2 (7.1)		
Sore throat	No		23 (85.2)	23 (82.1)	0.093	0.999
	Yes		4 (14.8)	5 (17.9)		
Scratchy throat	No		24 (88.9)	25 (89.3)	0.002	0.999
	Yes		3 (11.1)	3 (10.7)		
Cough	No		25 (92.6)	24 (85.7)	0.669	0.669
	Yes		2 (7.4)	4 (14.3)		
Hoarseness	No		24 (88.9)	26 (92.9)	0.262	0.669
	Yes		3 (11.1)	2 (7.1)		
Head congestion	No		26 (96.3)	27 (96.4)	0.001	0.999
	Yes		1 (3.7)	1 (3.6)		
Chest congestion	No		26 (96.3)	27 (96.4)	0.001	0.999
	Yes		1 (3.7)	1 (3.6)		
Feeling tired	No		25 (92.6)	23 (82.1)	1.351	0.422
	Yes		2 (7.4)	5 (17.9)		

No difference was observed between the probiotic and control groups, and no other adverse events were reported in the study.

## Discussion

We studied the efficacy of *Bacillus clausii* UBBC-07, supplemented along with whey protein for 60 days, on the PFAA levels in young male individuals who were involved in vigorous physical activities, including running, cricket, and football, or attending the gym on a daily basis. Compared to the control group, supplementation with *Bacillus clausii* UBBC-07 improved circulating total PFAAs, EAAs, and total and individual BCAAs including leucine, isoleucine, and valine. We observed that the improvement in the amino acid levels was higher at 30 days in comparison to 60 days of intervention. The reason behind this discrepancy could be poor adherence to prolonged intervention.

In previous studies, the effect of *Bacillus clausii *on amino acid absorption has been reported in combination with multi-strain probiotics. In a randomized, crossover, pilot clinical study, the impact of 15 days of an enzyme-probiotics blend containing *Bacillus clausii* (multispecies consortium including *Bacillus coagulans, Bacillus clausii, Bacillus subtilis, Lactobacillus acidophilus, and Lactobacillus plantarum*) with 30 g of pea protein on plasma amino acid levels was assessed. A significantly higher rate of absorption of a few EAAs and BCAAs including methionine (p=0.049), leucine (p=0.014), and isoleucine (p=0.053) was observed in the test group compared to the placebo [26]. A study by Jager et al. [5] reported increased plasma levels of BCAAs (leucine, isoleucine, valine) and other amino acids such as citrulline, glutamine, etc., with the consumption of one billion CFU *Bacillus coagulans* GBI-30 and whey protein for two weeks. In another study, the supplementation of two billion CFU* Bacillus coagulans* along with whey protein resulted in significantly improved circulating EAAs as well as BCAAs in physically-active males [7].

The mechanism of improvement in protein absorption with probiotic supplementation involves several key processes and interactions between probiotics and the digestive system. It is not clear yet whether probiotics affect protein absorption directly. However, their positive effects on gut health and function can indirectly support more efficient protein digestion and absorption. Probiotics can help maintain a balance of healthy intestinal microflora, which supports the integrity of the gut lining and reduces inflammation. They compete with harmful bacteria and pathogens and help to maintain a balanced gut environment, which supports better nutrient absorption. Probiotics can also enhance the host's digestive enzymes and their activity. This may aid in the breakdown of proteins and other nutrients and some can release exoenzymes involved in the digestion of proteins [27]. Probiotics can also influence gut permeability by promoting the production of mucins and other substances that strengthen the gut barrier.

The results of our study suggest an improvement in physical strength, as indicated by a significant change in the leg press and a trend of improvement in one RM deadlift and vertical jump with *Bacillus clausii* UBBC-07 consumption. The effect of *Bacillus clausii *UBBC-07 supplementation on muscle strength has not been reported before, but the supplemental efficacy of other probiotics in the *Bacillus* group has been demonstrated [7,28]. BCAAs play an important role in stimulating the rates of muscle protein synthesis and suppressing their breakdown, which promotes a net anabolic response in skeletal muscle. The intake of *Bacillus clausii *UBBC-07 was safe, and there were no adverse events in healthy individuals at dosages up to two billion CFU/day. There were no negative effects on the gastrointestinal system based on the assessment of abdominal pain, nausea, vomiting, stool irregularity, consistency, etc. Similarly, no negative effects on respiratory symptoms, including sneezing, runny and plugged nose, scratchy and sore throat, cough, head, and chest congestion, were evident. The results of our study are in concordance with previous studies. The limitation of our study is a lack of fecal microbiota analysis, which would have confirmed the balance of healthy intestinal microflora with probiotic supplementation [29].

## Conclusions

A significant improvement in circulating EAAs levels in the probiotic group suggests an enhancement of protein absorption with *Bacillus clausii *UBBC-07 supplementation. The effect of BCAAs, which enhance muscle strength, is evident in the significant improvement in leg press and a trend towards improvement in the deadlift and vertical jump in the probiotic group. This has positive implications for persons involved in sports. Future research should be directed towards understanding the improvement in the diversity of the gut microbiome with *Bacillus clausii* UBBC-07 supplementation. Also, a study with a modified age window would help determine the role of its supplementation along with whey protein in different age groups.

## Appendices

Appendix A 

**Table 4:** Exercise protocol

First day of the week (strength)	Third day of the week (back and biceps hypertrophy)	Fourth day of the week (leg hypertrophy)	Sixth day of the week (chest, shoulders, and triceps hypertrophy)
Bench press (7 sets), squats (7 sets), deadlift (7 sets), leg raise (3 sets), and weighted crunch (3 sets)	Pull-ups (7 sets), seated cable rows (7 sets), semi-supinated pull down with straight arm (7 sets), reserve barbell curl (7 sets), dumbbell curl (7 sets), and shrugs (6 sets)	Squats (6 sets), leg press (6 sets), leg extensions (7 sets), leg curl (7 sets), and calf raise (7 sets)	Bench press (6 sets), cable cross-over (6 sets), military press (5 sets), lateral raise (5 sets), triceps pushdown (5 sets), and French press (5 sets)

Appendix B

**Table 5:** PFAA levels in the probiotic and control groups (PP analysis) Data is represented as mean±SD and compared using repeated measures-ANOVA between the probiotic and control groups. A p-value <0.05 is considered significant. PP: per protocol; *B. clausii*: *Bacillus clausii; *PFAAs: Plasma free amino acids; BCAAs: Branched-chain amino acids; EAAs: Essential amino acids.

Amino acids (pmol/μl)	Group	Baseline	30 days	60 days	Time		Group		Time x Group	
					F-value	p-value	F-value	p-value	F-value	p-value
Total PFAAs	B. clausii	4009±1102	5014±1625	5122±1337	8.3	<0.001	5.6	0.022	6.6	0.002
	Control	4038±1180	4039±1002	4148±937						
Total BCAAs	B. clausii	392±93	570±300	542±192	11.3	<0.001	2.8	0.099	5.0	0.014
	Control	419±108.2	459±134	443±98						
Total EAAs	B. clausii	847±209	1170±499	1126±356	13.9	<0.001	2.5	0.120	5.5	0.008
	Control	896±209	976±271	949±185						
Aspartic acid	B. clausii	9.3±2.9	14.1±6.8	13.1±5.9	3.3	0.045	1.3	0.256	8.9	<0.001
	Control	11.5±5.6	9.8±3.2	11.3±6.7						
Glutamic acid	B. clausii	65.7±38.3	101±63.4	89.7±54.7	6.7	0.003	2.3	0.135	2.5	0.098
	Control	65.4±30.7	74±46.3	71.2±30.1						
Asparagine	B. clausii	66.3±22.3	81±32.5	76.8±31.7	9.7	<0.001	0.9	0.359	0.7	0.490
	Control	64.7±18	73.2±16.8	70.5±16.2						
Serine	B. clausii	127±39	169±73	162±59	6.2	0.005	1.4	0.238	4.8	0.014
	Control	137±48	137±45	142±42						
Glutamine	B. clausii	1458±471	1666±574	1792±575	3.0	0.053	7.6	0.008	4.6	0.013
	Control	1351±564	1247±438	1329±493						
Histidine	B. clausii	88.3±24.9	107.6±36	106.6±29.8	6.4	0.002	1.6	0.218	4.3	0.016
	Control	91.4±20.1	92.2±30	94.4±26.4						
Glycine	B. clausii	240±133	278±174	286±166	0.9	0.409	0.7	0.421	0.5	0.599
	Control	238±137	246±135	242±144						
Threonine	B. clausii	166±53	242±102	237±93	15.6	<0.001	1.9	0.169	4.9	0.009
	Control	177±56	204±81	191±63						
Arginine	B. clausii	78.9±26.8	82.9±52.7	101.7±47.9	8.6	<0.001	1.5	0.220	1.9	0.154
	Control	79±23.8	67.6±29	85.5±33.9						
Alanine	B. clausii	473±169	635±346	608±211	6.0	0.006	1.8	0.191	1.2	0.295
	Control	468±150	522±279	535±170						
Tyrosine	B. clausii	61.9±15.8	75.5±23	77.5±23.3	9.7	<0.001	2.9	0.095	2.4	0.100
	Control	61.9±11.3	65.4±17.3	68.1±14.5						
Cysteine	B. clausii	287±132	364±198	396±235	1.6	0.202	2.5	0.121	2.8	0.070
	Control	308±162	287±107	298±144						
Valine	B. clausii	261±64	357±174	345±144	8.8	0.001	2.0	0.162	8.8	0.026
	Control	275±77	295±91	289±73						
Methionine	B. clausii	20.8±4.2	24.7±9.4	25±6.6	4.0	0.028	0.8	0.373	6.0	0.005
	Control	22.6±3.3	21.7±5.3	22.6±4.2						
Tryptophan	B. clausii	37.6±7.6	44.5±13.6	46.1±15	4.9	0.010	0.2	0.665	3.8	0.027
	Control	41.5±8.1	41.4±9	42.4±8.1						
Phenylalanine	B. clausii	41±8.3	50.7±14.2	51.3±12.2	9.4	<0.001	1.2	0.283	3.6	0.032
	Control	43.6±8.2	46.1±12.8	45.9±9.2						
Isoleucine	B. clausii	38.6±8.3	59.9±34.8	57.8±24	14.4	<0.001	2.1	0.155	4.2	0.022
	Control	42.2±9	48.1±11.8	48.6±13.6						
Leucine	B. clausii	93±24	153±96	139±46	10.0	0.001	5.0	0.029	4.7	0.022
	Control	102±28	115±36	106±29						
Lysine	B. clausii	102±92	131±111	118±86	1.9	0.163	0.3	0.598	0.4	0.675
	Control	101±56	111±79	110±57						
Hydroxyproline	B. clausii	56±25.8	72.4±29.9	78.2±40.9	2.4	0.093	0.03	0.870	4.7	0.011
	Control	69.9±28.8	67.7±25	66±23.3						
Proline	B. clausii	238±120	305±218	315±164	1.9	0.161	0.1	0.822	3.9	0.025
	Control	288±170	265±117	280±119						

Appendix C 

**Table 6:** Strength of the participants at baseline and after 60 days between the probiotic and control groups (PP analysis) Data is represented as mean±SD and compared using repeated measures ANOVA between the probiotic and control groups. A p value <0.05 is considered significant. PP: per protocol; ANOVA: Analysis of variance; 1RM: One repetition maximum; *B. clausii: Bacillus clausii.*

Variable	Group	Pre-supplementation	Post-supplementation	Mean difference (95% CI)	Time		Group		Time x Group	
					F-value	P-value	F-value	P-value	F-value	P-value
Deadlift 1RM (kg)	B. clausii	77.71±23.96	92.5±27.61	14.79 (9.91, 19.67)	76.0	<0.001	1.4	0.238	3.0	0.092
	Control	70.56±28.37	80.48±33.16	9.92 (6.61, 13.23)						
	p-value	0.346	0.175	0.141						
Leg press 1RM (kg)	B. clausii	105.31±43.63	125.77±44.64	20.46 (14.73, 26.19)	78.0	<0.001	1.6	0.211	2.7	0.110
	Control	95.57±22.22	109.65±26.39	14.09 (8.44, 19.73)						
	p-value	0.339	0.137	0.045						
Bench press 1RM (kg)	B. clausii	62.81±17.66	75.31±15.62	12.5 (9.96, 15.04)	154.0	<0.001	0.02	0.897	0.6	0.446
	Control	64.24±22.47	75.28±22	11.04 (8.04, 14.04)						
	p-value	0.801	0.996	0.433						
Vertical jump (cm)	B. clausii	52.73±18.18	60.51±17.73	7.78 (5.49, 10.07)	67.1	<0.001	0.04	0.836	4.3	0.044
	Control	55.66±27	60.3±26.73	4.65 (2.5, 6.79)						
	p-value	0.660	0.974	0.109						
